# How do we choose the optimal length of flexible and navigable suction ureteral access sheaths (FANS): an EAU endourology and AUSET prospective multicenter analysis

**DOI:** 10.1007/s00345-026-06200-y

**Published:** 2026-01-19

**Authors:** Steffi Kar Kei Yuen, Daniele Castellani, Albert El Hajj, Bokye Soebhali, Deepak Ragoori, Khi Yung Fong, Chu Ann Chai, Chinnakhet Ketsuwan, Geavlete Petrisor, Mehmet Ilker Gokce, Karl Tan, Jia-Lun Kwok, Yi Quan Tan, Kremena Petkova, Palaniappan Sundaram, Lazaros Tzelves, Yadong Lu, Vigen Malkhasyan, Nariman Gadzhiev, Wissam Kamal, Luis Rico, Pablo Conteras, Giacomo Maria Pirola, Bhaskar Somani, Vineet Gauhar

**Affiliations:** 1 S.H. Ho Urology Centre, Department of Surgery, The Chinese University of Hong Kong, Hong Kong, China; 2 European Association of Urology Section of Endourology (ESEUT), Arnhem, The Netherlands; 3 Urology Unit, Azienda Ospedaliero-Universitaria Delle Marche, Via Conca 71, 60126 Ancona, Italy; 4 Department of Urology, American University of Beirut Medical Center, Beirut, Lebanon; 5 Department of Urology, Abdul Wahab Sjahranie Hospital Medical Faculty, Muliawarman University, Samarinda, Indonesia; 6 Department of Urology, Asian Institute of Nephrology & Urology, Irram Manzil Colony, Telangana Hyderabad, India; 7 Department of Urology, Singapore General Hospital, Singapore, Singapore; 8 Department of Surgery, Urology Unit, University of Malaya, Kuala Lumpur, Malaysia; 9 Division of Urology, Department of Surgery, Faculty of Medicine Ramathibodi Hospital, Mahidol University, 10400 Bangkok, Thailand; 10 Department of Urology, Carol Davila University of Medicine and Pharmacy, Bucharest, Romania; 11 Department of Urology, “Sf. Ioan” Clinical Emergency Hospital, Bucharest, Romania; 12 Department of Urology, Ankara University School of Medicine, Ankara, Turkey; 13 Department of Urology, Veterans Memorial Medical Center, Quezon City, Philippines; 14 Department of Urology, Tan Tock Seng Hospital, Singapore, Singapore; 15 Department of Urology, National University Hospital, 1E Kent Ridge Road, Tower Block, Level 8, 119228 Singapore, Singapore; 16 Division of Urology, Ng Teng Fong General Hospital, Singapore, Singapore; 17 Department of Urology and Nephrology, Military Medical Academy, Sofia, Bulgaria; 18 Department of Urology, Sengkang General Hospital, Singapore, Singapore; 19 2nd Department of Urology, Sismanoglio Hospital, National and Kapodistrian University of Athens, Athens, Greece; 20 Division of Surgery and Surgical Oncology, National Cancer Center Singapore, Singapore, Singapore; 21 Department of Urology, Moscow Urology Center, Botkin Hospital, Moscow, Russia; 22 Department of Urology, Saint Petersburg State University Hospital, Saint Petersburg, Russia; 23 Urology Unit, King Fahd General Hospital, Jeddah, Saudi Arabia; 24 Department of Urology, Hospital Alemán de Buenos Aires, Buenos Aires, Argentina; 25 IRCCS Multimedica, Ospedale San Giuseppe, Milan, Italy; 26 Department of Urology, University Hospital Southampton, NHS Trust, Southampton, UK; 27 Asian Institute of Nephrourology, AINU, Chennai, India

**Keywords:** Flexible and navigable suction ureteral access sheath, FANS, Flexible ureteroscopy, Kidney stones, Ureter, Length

## Abstract

**Purpose:**

To evaluate how to assess the optimal length of flexible and navigable suction ureteral access sheaths (FANS) to be used during flexible ureteroscopy (FURS) for kidney stones.

**Methods:**

A prospective multicenter study (16 centers, July 2024–January 2025) enrolled 226 adults with normal renal anatomy undergoing FURS with FANS for renal stones. Three preoperative measurements were analyzed: (1) *Subjective* on X-ray: T12–pubic symphysis, (2) *Objective* on CT: upper pole–pubic symphysis, and (3) *Dynamic* ureteral catheter length with retrograde pyelogram (RPG): upper pole–urethral meatus. Sheath length appropriateness (too short/correct/too long) was assessed intraoperatively using predefined criteria (complete calyceal access, ergonomics, need for ancillary techniques). Secondary outcomes included 30-day stone-free rates (SFR) and complications.

**Results:**

Sheath length was deemed correct in 63.7%, too short in 9.7%, and too long in 26.5% of cases. For both genders, dynamic measurement of ureteric catheter length from upper pole calyx to 5 cm beyond the urethral meatus demonstrated the strongest correlation with optimal length (*R* = 0.7). Gender-specific formulas for optimal FANS length were derived: Male: 0.52 × ureteral catheter length + 26 cm. Female: 1.2 × ureteral catheter length − 2.1 cm. Compensatory techniques for length discrepancies included assistant-held stabilization of FANS (28.3%) and telescoping of penis (3.5%). 96% of sheaths accessed all calyces. The 30-day SFR was 92.5% (Grade A: 79.2% zero fragments; Grade B: 13.3% fragments ≤ 2 mm). Complications were low: sepsis not requiring ICU admission (0.4%) and ureteric stricture (0.6%). Only 2.7% patients were planned for reintervention.

**Conclusion:**

The selection of an optimal FANS length is critical for optimizing outcomes in FURS. Dynamic RPG-ureteral catheter length measurement best predicts this, enabling gender-specific formulas for easy estimation. Our results provide valuable insights into the clinical relevance of preoperative measurements, intraoperative adjustments, and the impact of sheath length on procedural success and complications.

## Introduction

Flexible ureteroscopy (FURS) with flexible and navigable suction ureteral access sheaths (FANS) has revolutionized the management of nephrolithiasis [1]. FANS are characterized by their flexible non-traumatic tip that can be navigated past the ureteropelvic junction (UPJ) into the target calyces for suctioning of stone dust and fragments. Introduced in the 1970s, ureteral access sheaths (UAS) were typically placed below the UPJ and meant to serve as a conduit for flexible scope entry, re-entry, and regulation of irrigation and intrarenal pressure (IRP) [2]. Central to the success and safety of FURS are the appropriate selection and atraumatic deployment of UAS. The rate-limiting step in FURS is atraumatic deployment. A multitude of factors, including ureteral elasticity, compliance [3], orifice anatomy [4] and buckling or insertion force [5]are contributory. Whilst many studies have focused on strategies to optimise these, essential components include choosing the correct length and calibre to optimize surgical efficiency, minimize complications, and ensure favourable peri-operative patient outcomes.

As the distal 10–12 cm of FANS is flexible and is designed to be able to navigate the entire pelvicalyceal system (PCS), the FANS needs to be placed across the UPJ. Hence, if too short, parts of the PCS, especially the lower pole, may be inaccessible; if too long, the sheath can protrude much beyond the genitalia, mandating assistance to stabilise the sheath, especially when using the scope and sheath together, a phenomenon seen in early experience of using adult FANS in paediatric cases, for which no dedicated FANS exists. Additionally, simultaneous aspiration of dust, debris, and fragments may be ineffective hence defeating its primary function [6]. Preminger et al. [7] have shown that a conventional UAS placed close to the UPJ best reduces IRP. This is why, typically, a 150 –180 cm tall person requires a 35–40 cm conventional UAS to reach the renal pelvis. Several studies have examined optimal sheath diameter [8] and scope type [9], endoscope-sheath diameter [10], optimal deflection techniques [11] and even flow dynamics on particle size [12] to maximise the best outcomes of FURS with FANS but no study has assessed its optimal length.

To our knowledge, no technique has been described for how to measure the correct UAS length. Due to paucity of literature on how to choose a correct length of FANS, we took insights from stent studies and stent-based surveys on perioperative and intraoperative measurements that help choose the optimal length critical to avoid stent migration (too short) and stent discomfort (too long) [13–16].

Our study primarily aimed to identify an easy-to-use strategy and an easy-to-use formula to choose the optimal FANS length. Our secondary outcomes were to report intraoperative and 30-day postoperative outcomes.

## Materials and methods

Sixteen centers worldwide contributed prospective data on adult kidney stone patients who underwent FURS with FANS from July 2024 to January 2025. Anonymized data from consented patients were maintained in the registry by the primary site: Asian Institute of Nephro-Urology, Hyderabad (AINU-EC/28/2023).

Baseline and operative characteristics were gathered. Inclusion criteria were patients aged ≥ 18 years with normal renal anatomy who had FURS using FANS for solitary/multiple renal stones. Children and patients who had abnormal renal anatomy or incomplete data were excluded.

The following measurements were taken by operating urologist prior to surgery on screen at the time of surgery on stone protocol 2 mm cut NCCT DICOM as per the designated measurement schema to maintain uniformity.

**Fig. 1:**
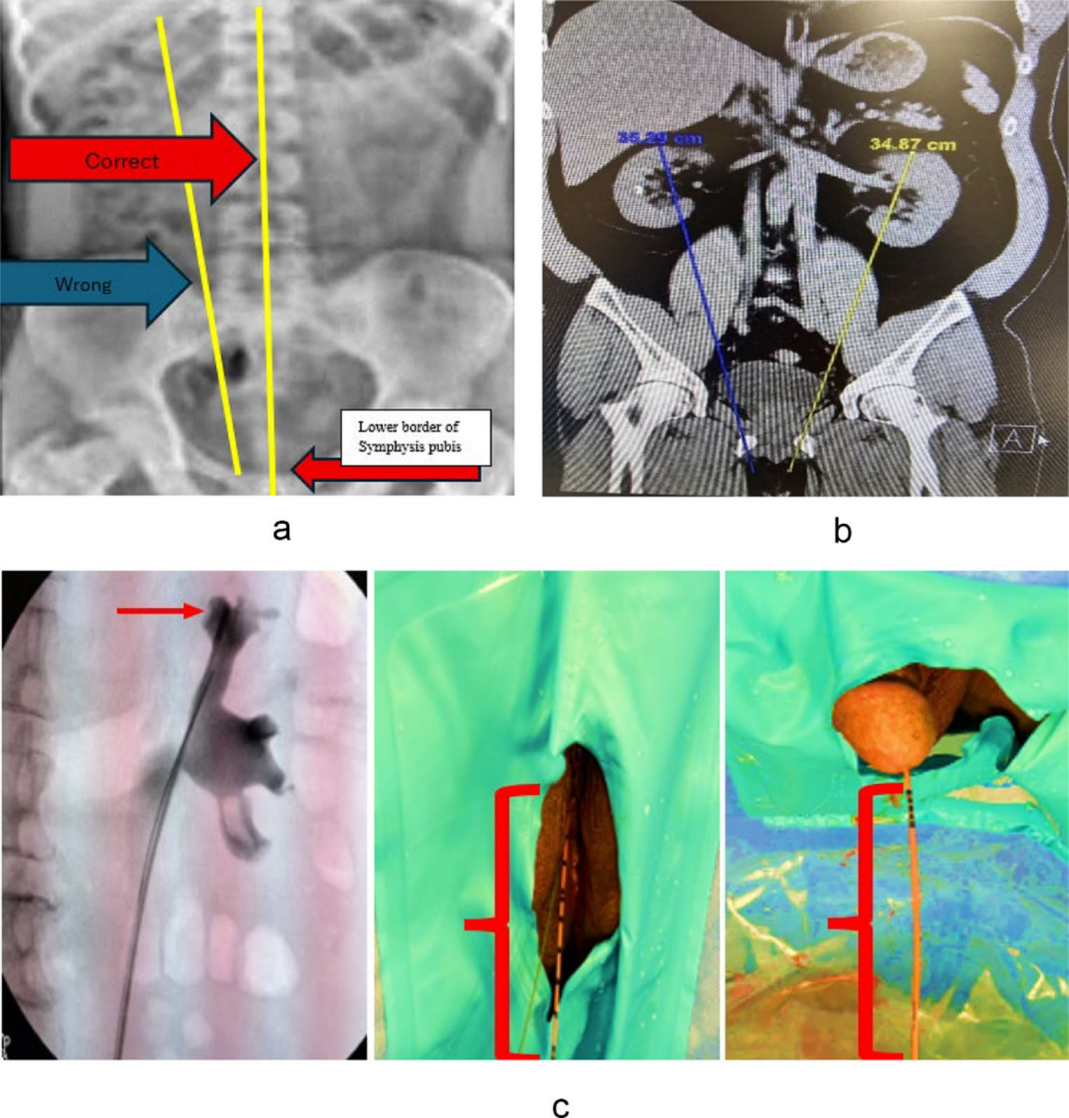
**a** Subjective measurement on X-ray KUB from T12 to pubic symphysis. **b** Objective measurement on CT KUB coronal view from the most convex point of the upper pole of the kidney to pubic symphysis: Choose a Coronal view of the KUB that must include upper border of the most convex point of the upper pole of the kidney. Then draw a line towards the lower border of the pubic rami as shown. **c** Real-time dynamic measurement from the upper pole to urethral meatus from on-table retrograde pyelogram (RPG) via ureteric catheter placed all the way to the uppermost part of upper pole and externally 5 cm beyond the meatus

Before patient enrolment and to maintain uniformity in measurements, all surgeons were clearly educated and trained either online or in person by two of the authors (VG/SY). Surgeons subjectively chose the length of FANS on their own choice. Postoperatively, surgeons scored the sheath as too short, too long, or appropriate as per the criteria stated below. Surgeons were also asked to grade their experience of FANS usage at the end of each case using a 5-point Likert-type scale (1 = excellent; 2 = very good; 3 = good; 4 = average; 5 = difficult).

### Defining the optimal FANS sheath length

**Fig. 2:**
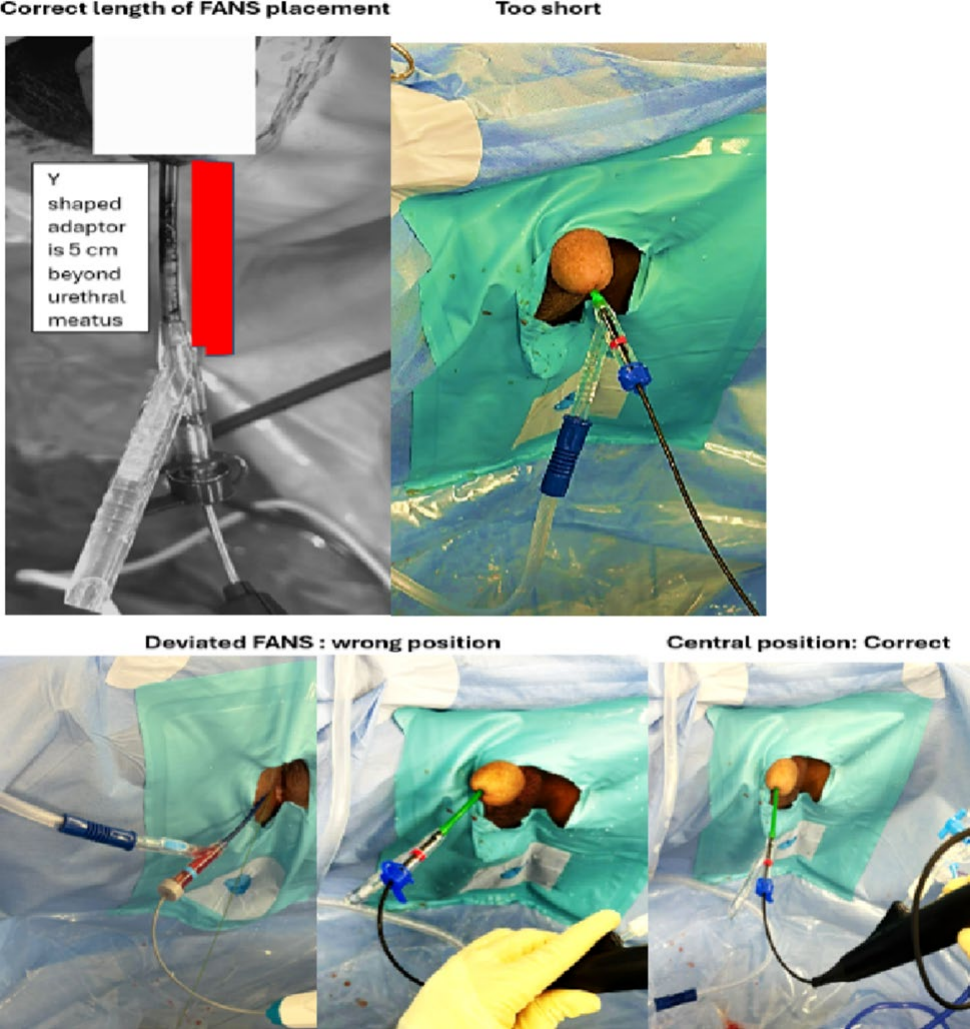
Illustration of correct length and inappropriate FANS length

Three measurements (Fig. 1a–c) were made, namely subjective, objective, and dynamic as defined above. Surgeons would grade length appropriateness as per Fig. 2 upon conclusion of surgery.

Correct length is defined by 3 criteria from consensus by authors:

- The flexible and deflectable part of the sheath can be deflected into all parts of the kidney, including the lower pole.
- No physical hindrance to the suction tubing application to the Y connector due to the patient’s genitalia.
- There is no need for telescoping the sheath into the genitalia to access all parts of the PCS. Telescoping is defined as pushing the sheath distal end all the way into the genitalia with or without distortion of the genitals.

Too long. Any 1 criterion of the following:

- The distal end of the sheath extends more than 5 cm from the external meatus.
- The sheath needs an assistant to stabilize.

Too short. Any 1 criterion of the following:

- The genitalia need to be telescoped in a male or sheath abutting the external genitalia in a female.
- The sheath cannot access the lower pole.

Only 7.5Fr single-use flexible ureteroscopes and 10/12Fr size FANS of any brand/ manufacturer were used. Irrigation flow rate of 100 ml/min and aspiration pressure of 100mmHg were recommended. Irrigation mode and flow rates, suction device, and pressure were charted. 200 micron laser fibers were uniformly utilized. Laser choice, perioperative decisions, and exit strategy were at the respective surgeons’ own discretion based on their operative experience and resource availability.

Operative time was recorded as the time from the start of cystoscopy to the exit strategy (stent, ureteric catheter, or nil drainage). Postoperative outcomes of interest included complications within 30 days, such as bleeding, transfusion, ureteric injury, PCS injury, infective complications, persistent hematuria, and loin pain score; as well as 30-day stone-free rates and re-intervention. The long-term outcome of interest was ureteric stricture at 3 months, assessed using a CT urography.

All patients had a preoperative XR-KUB for measurement and a preoperative NCCT scan done within 4 weeks before surgery to estimate stone volume and measurements. The stone volume before surgery was manually calculated from the CT scan using the bone window and by measuring the stone diameter along 3 axes and applying the ellipsoid formula (length × width × depth × π × 0.167).

A postoperative NCCT scan at 4 weeks was performed to assess stone-free status and residual fragments (RF) graded as below.

Grade A: 100% stone-free, zero residual fragments (ZRF); no fragments visible on the CT scan.

Grade B: single RF not more than 2 mm in maximum diameter.

Grade C: Single RF 2.1–4 mm in maximum diameter.

Grade D: Single RF > 4 mm or multiple residual fragments (RF) of any size.

All patients had a 3 month follow up post primary intervention.

### Statistical analyses

All statistical analyses were performed using R Statistical language, version 4.3.0 (R Foundation for Statistical Computing, Vienna, Austria), with *p* < 0.05 indicating statistical significance. Continuous variables were described using median and interquartile range, while categorical variables were described using absolute numbers and percentages. The Shapiro-Wilk test was used to assess for normality. Logistic regression was performed for correlations between subjective, objective, and dynamic measurements and FANS length. The Pearson correlation coefficient (R) was employed to assess the correlation between preoperative measurements and optimal sheath length, where *R* ≥ 0.7 indicates a strong correlation, while 0.3 ≤ *R* < 0.7 a moderate correlation and *R* < 0.3 weak/negligible correlation.

## Results

226 patients were included. Baseline and operative characteristics are tabulated in Table 1. The median age was 54 years; 57.1% were male. Loin pain was the predominant symptom (68%). 46.2% were pre-stented. Most stones were located in the lower pole (41.9%), followed by the middle pole or interpolar region (37.2%), with a median stone volume of 700mm^3^ and Hounsfield unit of 1120. The mean irrigation flow rate of 80 ml/min, minimal and maximum suction pressure of 90mmHg and 150mmHg, respectively.

### Measurements and correlation

Preoperative median subjective measurement of X-ray KUB from T12 vertebrae to pubic symphysis was 32.6 cm [29.1, 36.0]; median objective measurement from upper pole to pubic symphysis on CT was 32.3 cm [28.4, 35.2]; median dynamic measurement from upper pole to urethral meatus on retrograde pyelogram (RPG) by ureteric catheter was 40.0 cm [34.0, 44.0] (Table 2 ).

### Clinical appropriateness of sheath length selection

Sheath lengths utilized ranged from 35 cm to 55 cm. FANS length was deemed appropriate in 63.7%, while 26.5% perceived it as too long and 9.8% too short. No case required change of FANS due to length discrepancy. 28.3% of patients with length discrepancy required assistance with FANS intraoperatively, and 3.9% required excessive torque of the scope shaft (3.9%). Nevertheless, 96% of the sheaths successfully accessed all parts of the PCS. The mean Likert score for ease of suction, manipulation, and visibility during FANS operation was rated 1,1,2, respectively, reflecting overall surgeon satisfaction.

**Fig. 3:**
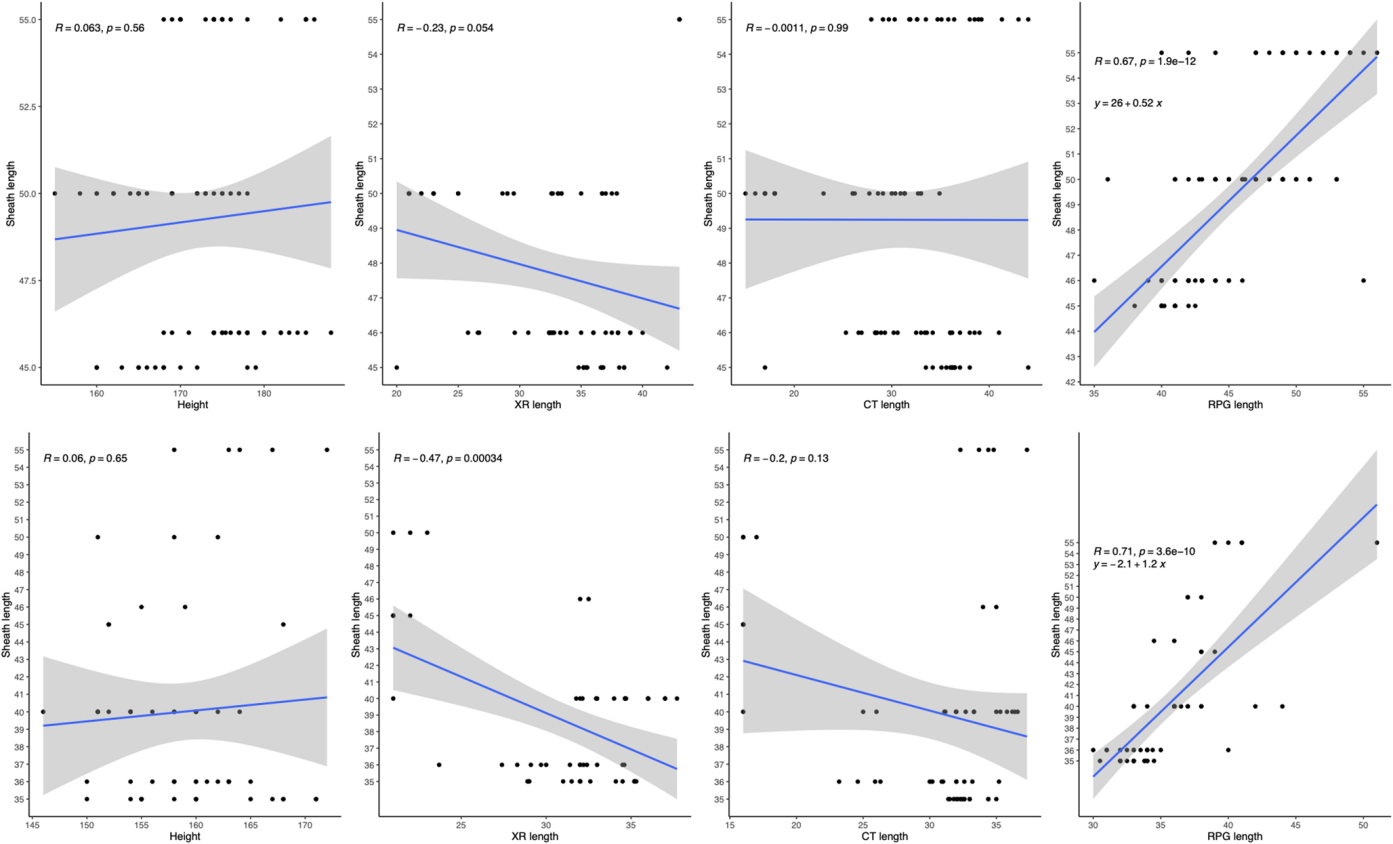
Regression line graphs comparing height, X-ray length, CT length, RPG-ureteral catheterlength against sheath length in female patients (top row) and male patients (bottom row) (left toright)

The strongest correlation between preoperative measurements and optimal sheath length was observed with the RPG method (upper pole to urethral meatus; *R* = 0.7 for both genders). Whilst R values of height, X-ray length, and CT were 0.063, -0.23, -0.0011, respectively, in males; 0.06, -0.47, -0.2 in females (Fig. 3).

The real-time dynamic measurement of the length of ureteric catheter placed from upper pole calyx to 5 cm beyond the urethral meatus demonstrated the strongest correlation with optimal length (*R* = 0.7). Gender-specific formulas were derived:

- Optimal male FANS length: 0.52 × ureteral catheter length + 26 cm.
- Optimal female FANS length 1.2 × ureteral catheter length − 2.1 cm.

### Postoperative outcomes and stone-free rates

At 30 days, ZRF (Grade A SFR) was seen in 79.2%, Grade B 13.3% and a low complication rate, namely 0.4% sepsis and 2.7% reintervention were reported. Basket utilization was noted in 13.3%.

Grade C and D SFR were seen in 4.9% and 2.7% respectively. These were mainly in suboptimal sheath lengths, requiring surgeon adjustments to complete the procedure.

Only one patient (0.6%) developed ureteral stenosis (0.6%) at 3 months, warranting further causal investigation. The patient’s 1.8 cm upper pole renal stone was treated with a 10/12Fr FANS of 46 cm, which was reported to be too long by the operating surgeon (6 cm measuring from the genitalia to the end of the FANS y-connector). There was an intraoperative PCS injury during FANS manipulation managed conservatively by prolonged postoperative stenting of 30 days. Hydronephrosis was noted upon stent removal. Ureteral stenosis was managed with ureteral dilatation and stenting(Table 3).

## Discussion

Compared to conventional UAS, FANS is becoming widely popular as the sheath of choice [17, 18] in FURS, as it improves the single-stage stone-free rate and even attains ZRF status when used diligently with due care given intra-operatively to safely examine all calyces and aspirate any dust and fragments. This makes it imperative to not only choose the correct diameter to avoid access sheath injuries but also deploy the correct length, as failure to do so can cause PCS injuries, genitalia injuries, ineffective suction and aspiration, need for additional accessories to remove inaccessible fragments and missed fragments due to inability to reach deeper parts of the kidney, such as the lower pole. Thus, negatively the trifecta of surgical outcomes surgeons aims for, which makes this a true game-changer. Notably, surgeons who did have length discrepancies, both too short or too long, reported issues as mentioned before.

Intraoperative problems due to suboptimal sheath length can be a sheath deemed “too long” (26.5% of cases) which may compromise ergonomics, increase the risk of displacement, or impede scope maneuverability. Conversely, a “too short” sheath risks incomplete renal access, necessitating compensatory maneuvers that may prolong operative time.

An inappropriate sheath length can be hard to use without assistance, as seen when adult FANS were applied in paediatric cases [19] or may risk injury to genitalia due to telescoping of same, and can lead to over-torquing or scope damage. If most of the scope shaft remains outside the excess sheath, the scope may not reach deeper kidney areas, especially the lower pole. Using the wrong size sheath also wastes resources and may hinder laser lithotripsy or suction evacuation of fragments.

The aforementioned reasons are mainly speculative, hearsay, or isolated reported events amongst surgeons. Hence, there is a need to study how to choose the correct sheath length and mitigate an adverse event during FURS.

To our knowledge, this is the first practical, real-world study that aims to determine an appropriate FANS length. Our RPG-based correlations support that dynamic intraoperative measurements outperform static imaging for length selection. This anatomical approach likely accounts for the entire ureteral course, including dynamic changes of bladder filling and urethral length, which static imaging (e.g., CT or X-Rays) may underestimate the overall and right length. As noted in studies that unsuccessfully looked at anthropometric measurements [15, 16], we too decided against the same. Instead, xyphoid process to pubis lengths had a better correlation in stent studies, and this was modified and adopted as the RPG-ureteral catheter length.

This study is limited by the fact that there is no preceding similar study to compare and validate it. Despite our best efforts to streamline and simplify the definitions of correct and incorrect sheath measurement, there remains potential subjectivity in surgeon-reported Likert scores and interpretation. Inter-surgeon variability in RPG/CT measurement techniques across centres may introduce bias.

Furthermore, this study was based on the feedback and personal experience of experienced surgeons who first participated in the Global FANS study [1] and criteria were framed based on their personal experiences, which were unreported in the literature. Nonetheless, not choosing the correct FANS length and issues associated with the same were clearly reflected in the global FANS study in pediatric cases [19, 20], where an assistant was needed to hold the sheaths that extended too far beyond the meatus and reportedly also caused ergonomic issues.

Whilst our study revealed that due to sheath length discrepancy, surgeons had to telescope the penis, sought assistant help, or applied excessive torque on the scope shaft. This was not causally related to any genital injuries, scope breakages, sheath malfunction, or change. Further, surgeons were able to overcome these issues and successfully access all calyces in 96.3% of cases. As a consequence of incorrect sheath length, basket utilization was higher than reported in the literature [21]. This anatomical approach likely accounts for the entire ureteral course, including dynamic changes of bladder filling and urethral length, which static imaging (e.g., CT or X-Rays) may underestimate the overall length. The use of compensatory techniques—such as assistant-held sheath stabilization (28.3%) and excessive shaft torque (3.9%)—highlights the practical challenges when preoperative measurements and estimation do not perfectly align with intraoperative needs. Despite these discrepancies, 96% of sheaths successfully accessed all parts of the pelvicalyceal system, underscoring the adaptability of surgeons in mitigating length-related limitations.

This study is also limited by the generalization of application in normal adult renal anatomy. We are unsure if this will hold good in patients with anomalous, altered physical or PCS anatomy, thus limiting generalizability to complex cases. Additionally, as surgeons used sheaths from different manufacturers, inter-brand differences in actual versus quoted lengths could introduce bias. Unless validated externally, our results are a provisional attempt; nevertheless, we can infer that preoperative imaging cannot accurately estimate the correct sheath length, potentially avoiding unnecessary imaging. We hope that refinements in sheath design could enable manufacturers to tailor equipment to specific populations, an example being the paediatric age group, where it has been reported that using adult instruments in small children poses challenges and impacts surgical outcomes [22, 23]. If validated, these findings can be targeted to population-specific studies, as demonstrated in prior stent-based research in Chinese and Korean populations [14].

## Recommendations

1. Operative planning: Advocate RPG-based length measurement using our gender-specific formulas to guide initial sheath selection.
2. Training of FANS utilization: Equip surgeons with compensatory techniques (e.g., torque navigation, assistant stabilization) for unavoidable mismatches.
3. Technology Development: Manufacturers should offer expanded length options (e.g., 5-cm increments) and population-specific designs (e.g., pediatric sheaths).
4. Future Research: Validate our findings in larger and diverse cohorts and assess impacts on operative efficiency, radiation exposure, and long-term ureteral health.

Surgeons should also receive training in compensatory techniques to address unavoidable length mismatches.

**Table 1:** Baseline characteristics

	*N* = 226
Age	54 [43, 64]
Male sex	129 (57.1)
Height, cm	165 [160, 175]
BMI, kg/m^2^	26.1 [24.0, 29.0]
ASA	
1	93 (41.2)
2	95 (42.0)
3	37 (16.4)
4	1 (0.4)
Anticoagulant/antiplatelet use	30 (13.3)
DM	47 (20.8)
HTN	60 (26.5)
IHD	20 (8.8)
First-time stone former	119 (52.9)
Presentation	
Hematuria	29 (12.9)
Pain	153 (68.0)
Fever	7 (3.1)
Incidental stone	36 (16.0)
Urine culture positive	62 (27.6)
Prestented	104 (46.2)
Reason for those prestented	
Failure to access ureter	43 (42.6)
Routine, for staged RIRS	12 (11.9)
Emergency pre-stenting for sepsis	46 (45.5)
Right-sided stone	92 (40.7)
Hounsfield units	1120 [940, 1343]
Stone volume, mm^3^	700 [400, 2100]
Stone location	
Upper pole	39 (18.1)
Middle pole/interpolar	80 (37.2)
Lower pole	90 (41.9)
Multiple locations	6 (2.8)
Preoperative measurements, cm	
T12 to pubic symphysis (XR)	32.6 [29.1, 36.0]
Upper pole to pubic symphysis (CT)	32.3 [28.4, 35.2]
Upper pole to urethral meatus (RPG)	40.0 [34.0, 44.0]
Sheath length deemed suitable (cm)	
35	18 (8.0)
36	26 (11.5)
40	42 (18.6)
45	39 (17.3)
46	37 (16.4)
50	37 (16.4)
55	18 (8.0)

*BMI* body mass index; *ASA* American Society of Anesthesiologists; *DM* diabetes mellitus; *HTN* hypertension; *IHD* – ishemic heart disease; *XR* X-Rays; *CT* computer tomography; *RPG* retrograde pyelogram

**Table 2:** Operative characteristics

	*N* = 226
Procedure under general anesthesia	187 (82.7)
Laser	
LPHL	33 (14.6)
HPHL	79 (35.0)
HLM	3 (1.3)
TFL	98 (43.4)
ThuYAG	13 (5.8)
Median dusting settings, J/Hz	0.5/25
Median popcorning settings, J/Hz	1.0/15
Irrigation device	
Pressurized bag with Manchester cuff	67 (33.7)
Endomat (mechanical)	10 (5.0)
Endoflow (vacuum chamber + Traxer flow)	15 (7.5)
Irrigation bag (gravity alone)	51 (25.6)
Others	56 (28.1)
Irrigation flow, ml/min	80 [25, 106]
Median min/max suction settings, mmHg	90/150
Total operation time, min	47 [35, 71]
Stone clearance modality	
Dusting	80 (35.4)
Popcorning	36 (15.9)
Fragmentation	30 (13.3)
Combination	80 (35.4)
Basketing to aid stone removal	30 (13.3)
Sheath change due to length mismatch	0
Sheath able to access all of kidney	217 (96.0)
FANS length judgement, Likert scale*	3 [3, 3]
FANS length judgement, category	
Too short	22 (9.7)
Too long	60 (26.5)
Correct	144 (63.7)
Additional techniques to aid suction in view of length discrepancy	
Excessive torque causing shaft damage	8 (3.9)
Need for telescoping the penis	8 (3.5)
Need for assistant help to hold sheath due to inappropriate sheath length	64 (28.3)
Intraoperative stone free assessment	
Ability to manipulate sheath to achieve 100% stone free	138 (61.1)
Exit strategy	
Double J Stent (1–2 weeks)	135 (59.7)
Overnight ureteric catheter only	43 (19.0)
Stentless	48 (21.2)
Likert scale rating by surgeon, mean ± SD	
Ease of suction	1.0 [1.0, 2.0]
Manipulation	1.0 [1.0, 3.0]
Visibility	2.0 [1.0, 4.8]

All sheath sizes 10/12 Fr with 7.5 Fr FURS

*LPHL* low pole Holmium laser; *HPHL* high power Holmium laser; *HLM* Holmium: YAG laser with moses technology; *TFL* thulium fiber laser; *ThuYAG* thulium yttrium aluminium garnet; *FANS* flexible and navigable suction ureteral access sheath; *SD* standard deviation

*1: too short, cannot reach all parts even with active deflection, 2: too short but can push the sheath into all parts without scope (passively) by telescoping the genitalia, 3: ideal length, 4: too long, hindering scope re-entry and ergonomics, 5: too long, weight of extended part requires assistance or holding in place for fear of slipping out

**Table 3:** Postoperative outcomes

	*N* = 226 ( %)
Case abandoned	0
Postoperative transfusion (CD2)	0
Ureteric injury	0
PCS injury (CD2)	1 (0.6)
Median postoperative loin pain score	2 [1, 3]
Fever < 38.C resolved with just antibiotics (CD2)	6 (2.7)
Sepsis (CD4)	1 (0.4)
Hematuria prolonging stay (CD2)	0
AVM requiring embolization (CD3)	0
Hospital stay, days	2 [1, 2]
Residual fragment grading on 30-day NCCT scan	
A (zero residual fragments)	179 (79.2)
B	30 (13.3)
C	11 (4.9)
D	6 (2.7)
Stone free (A + B) – no > 2 mm fragments	210(92.5)
Reintervention for stone	6 (2.7)
Ureteric stricture/stenosis at 3 months on CTU	1 (0.6)

*CD* Clavien Dindo; *NCCT* non contrast computer tomography; *CTU* computer tomography urography

## Conclusion

The selection of an appropriate ureteral access sheath length is critical for optimizing outcomes in flexible ureteroscopy with FANS. Dynamic RPG-ureteral catheter measurement best predicts optimal FANS sheath length, enabling gender-specific formulas for easy estimation. Our multicenter study provides valuable insights into the clinical relevance of preoperative measurements, intraoperative adjustments, and the impact of sheath length on procedural success and complications. Developing institution-specific nomograms or predictive algorithms incorporating RPG data could further refine accuracy.

## Data Availability

The data that support the findings of this study are available from the corresponding author upon reasonable request.
